# Liquid-phase microextraction of aromatic amines: hollow fiber–liquid-phase microextraction and parallel artificial liquid membrane extraction comparison

**DOI:** 10.1007/s00216-023-04579-w

**Published:** 2023-02-23

**Authors:** Nerea Lorenzo-Parodi, Wiebke Kaziur-Cegla, Astrid Gjelstad, Torsten C. Schmidt

**Affiliations:** 1 Instrumental Analytical Chemistry, University of Duisburg-Essen, Universitätsstrasse 5, 45141 Essen, Germany; 2 Department of Pharmacy, University of Oslo, Blindern, P.O. Box 1068, 0316 Oslo, Norway; 3 Centre for Water and Environmental Research, University of Duisburg-Essen, Universitätsstrasse 5, 45141 Essen, Germany; 4 IWW Water Centre, Moritzstrasse 26, 45476 Mülheim an der Ruhr, Germany

**Keywords:** Hollow fiber-liquid-phase microextraction (HF-LPME), Parallel artificial liquid membrane extraction (PALME), Liquid–liquid extraction, Aromatic amines, GC–MS, Urine

## Abstract

Aromatic amines (AA) are carcinogenic compounds that can enter the human body through many sources, one of the most important being tobacco smoke. They are excreted with the urine, from which they can be extracted and measured. To that end, hollow fiber-liquid-phase microextraction (HF-LPME) and parallel artificial liquid membrane extraction (PALME) were optimized for the analysis of representative aromatic amines, as alternatives to liquid–liquid extraction (LLE). Relevant extraction parameters, namely organic solvent, extraction time, agitation speed, and acceptor solution pH, were studied, and the two optimized techniques—HF-LPME: dihexyl ether, 45 min, 250 rpm, and pH 1; PALME: undecane, 20 min, 250 rpm and pH 1—were compared. Comparison of the optimized methods showed that significantly higher recoveries could be obtained with PALME than with HF-LPME. Therefore, PALME was further validated. The results were successful for nine different AA, with regression coefficients (*R*^2^) of at least 0.991, limits of detection (LOD) of 45–75 ng/L, and repeatability and peak area relative standard deviations (RSD) below 20%. Furthermore, two urine samples from smokers were measured as proof of concept, and 2-methylaniline was successfully quantified in one of them. These results show that PALME is a great green alternative to LLE. Not only does it use much smaller volumes of toxic organic solvents, and sample—enabling the study of samples with limited available volumes—but it is also less time consuming and labor intensive, and it can be automated.

**Graphical Abstract:**

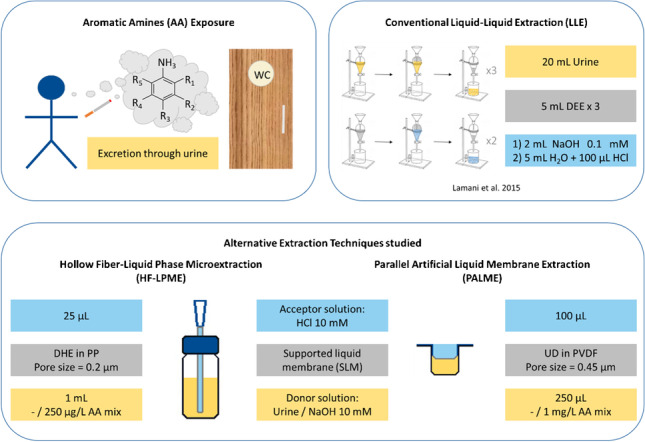

**Supplementary Information:**

The online version contains supplementary material available at 10.1007/s00216-023-04579-w.

## Introduction

Aromatic amines (AA) are highly toxic compounds, some of which are officially classified as carcinogenic [1]. They are used in several industries, such as during the manufacture of pharmaceuticals, pesticides, dyes, rubber, or resins [2]. Another important source of exposure to humans is tobacco smoke [3]. When the smoke is inhaled, aromatic amines enter the bloodstream and are transported through the body and metabolized until they reach the bladder. There, their metabolites can either be excreted with the urine or form DNA and protein adducts that can induce bladder cancer [2, 4, 5].

Urine samples, like most biological samples, require thorough sample preparation prior to their analysis in order to minimize potential interferences with matrix compounds, such as proteins, peptides, or salts, present in the samples [6]. The most commonly used clean-up technique for the extraction of compounds from aqueous samples is liquid–liquid extraction (LLE) [7]. However, LLE presents several disadvantages, such as being time consuming and labor intensive, and, therefore, is prone to human errors. Furthermore, it typically uses high amounts of organic solvents, which are often highly toxic, and needs relatively large sample volumes, which is especially critical in situations where sample volume is limited, like with archived urine samples. To overcome these drawbacks, two liquid-phase microextraction (LPME) techniques were evaluated as alternatives: hollow fiber-LPME (HF-LPME) and parallel artificial liquid membrane extraction (PALME).

Hollow fiber-LPME (HF-LPME) was developed in 1999 by Pedersen-Bjergaard and Rasmussen [8, 9] and parallel artificial liquid membrane extraction (PALME) was first introduced in 2013 by Gjelstad et al. as an alternative to HF-LPME [10]. Both methods are based on creating a supported liquid membrane (SLM) that aids on the extraction process [9]. SLM-based techniques, like HF-LPME and PALME, offer a green alternative to LLE thanks to the much smaller volumes of organic solvent needed, which contributes to reducing the costs and the environmental footprint per sample. They have a simpler workflow than LLE, not only for two-phase extractions, where the acceptor solution is the same organic solvent used for the SLM, but especially for three-phase extractions, where it is an aqueous solution, enabling the extraction into an organic solvent and back extraction into an aqueous solution to be carried out simultaneously. Moreover, they typically extract less matrix interferences thanks to the extra physical barrier, i.e., the solvent-filled porous membrane. Furthermore, the use of disposable fibers/well plates eliminates the possibility of carry-over and the need for cleaning/regeneration [9]. These characteristics make SLM-based techniques especially suitable for complex biological samples, like blood or urine.

HF-LPME offers a high flexibility regarding the donor and acceptor volumes, and therefore on the enrichment factors observed [9]. In recent years, automation has become one of the trends of current research regarding LPME, leading to several successful automation attempts, as summarized by [11]. Unfortunately, there are no commercially available fibers for HF-LPME yet [9].

There is commercially available equipment suitable for PALME, which facilitates a semi-automatic or fully automatic extraction and a successful validation. At the same time, it limits the range of sample volumes that can be used. Because the membranes available are made of polyvinylidene fluoride (PVDF) instead of the more inert polypropylene used for the fibers, non-specific binding for some substances can be observed, leading to non-linear calibration curves [12]. Less time is needed to set up the extraction and more samples can be processed simultaneously, making it more user-friendly and enabling higher sample throughputs [9].

The aim of this paper was to study the suitability of HF-LPME and PALME for the analysis of aromatic amines in urine. This is, to the best of our knowledge, the first paper in which PALME was used for these analytes and the two LPME techniques were compared with each other.

## Materials and methods

### Chemicals and reagents

The aromatic amines used (Table 1) were purchased from Sigma-Aldrich (Steinheim, Germany), except for 4-chloro-2-methylaniline and 3-chloro-2,6-dimethylaniline which were purchased from Fluka (Darmstadt, Germany) and Alfa Aesar (Karlsruhe, Germany), respectively.

**Table 1:**
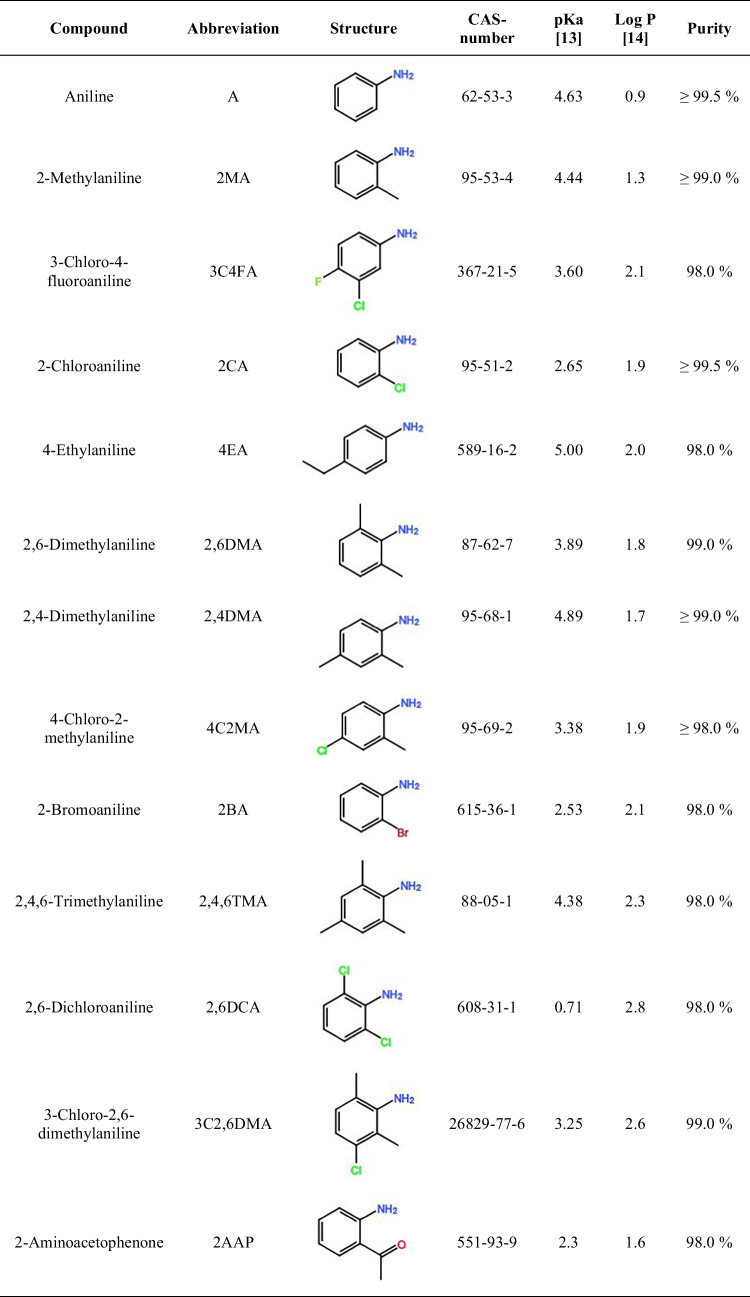
List of aromatic amines used with their corresponding abbreviation, structure, CAS-number, pKa value of the corresponding anilinium ion, log *P* value for the neutral compound, and purity

For the LPME optimization, the solvents dodecyl acetate (97%, abbreviated DDA), undecane (≥ 99%, UD), and dihexylether (97%, DHE) were purchased from Sigma-Aldrich, and 2-octanone (98%, 2O) was purchased from Alfa Aesar. Concentrated hydrochloric acid (ACS reagent, 37%, HCl) and sodium hydroxide (98%, NaOH) were purchased from Bernd Kraft (Duisburg, Germany).

During the derivatization, hydriodic acid (ACS reagent, unstabilized, 55%), sodium nitrite (99%), and Alizarin red S (98%) obtained from Sigma-Aldrich, and sodium sulfite (puriss. p.a., ACS reagent, RT, ≥ 98%) and sulfamic acid (T, ≥ 99%) from Fluka were used.

Diethyl ether (DEE) and HPLC grade methanol were purchased from Fisher Scientific (Schwerte, Germany), and ultrapure water was obtained from a PureLab Ultra water system from ELGA LabWater (Celle, Germany).

### Preparation of stock and standard solutions

Stock solutions were prepared for each aromatic amine studied, by weighing 10 mg of the pure substance in a 10-mL volumetric flask and diluting with methanol to a final concentration of 1 g/L. An intermediate stock containing 50 mg/L of each analyte (standard mix) was prepared monthly and further diluted in methanol to 2 mg/L and 0.2 mg/L. The solutions were kept refrigerated at 8 °C while not in use.

### Sample preparation

In order to achieve the same theoretical acceptor concentration, to minimize the influence of other factors in the results and facilitate the comparison between the techniques, donor concentrations of 250 µg/L and 1 mg/L were used for the HF-LPME and PALME optimization experiments, respectively. The donor solutions were prepared by spiking 10 mM NaOH (pH = 12) with the 50 mg/L standard mix.

Because of the significantly better performance of PALME, only this technique was further used for validation experiments. For the calibration curve experiments, samples with concentrations from 100 to 1200 ng/L in ultrapure water, which were alkalized with NaOH until pH 13.5, were used. As a proof of concept, two real samples from donors (smokers) were measured.

The samples were derivatized, and analyzed with solid-phase microextraction-gas chromatography-mass spectrometry (SPME–GC–MS), and the performance of the system was checked by measuring control samples on a weekly basis. Samples with a concentration of 2.5 mg/L aromatic amines and pH 2 were prepared, and aliquots of 100 µL were derivatized and analyzed with SPME–GC–MS.

#### Extraction

**Fig. 1:**
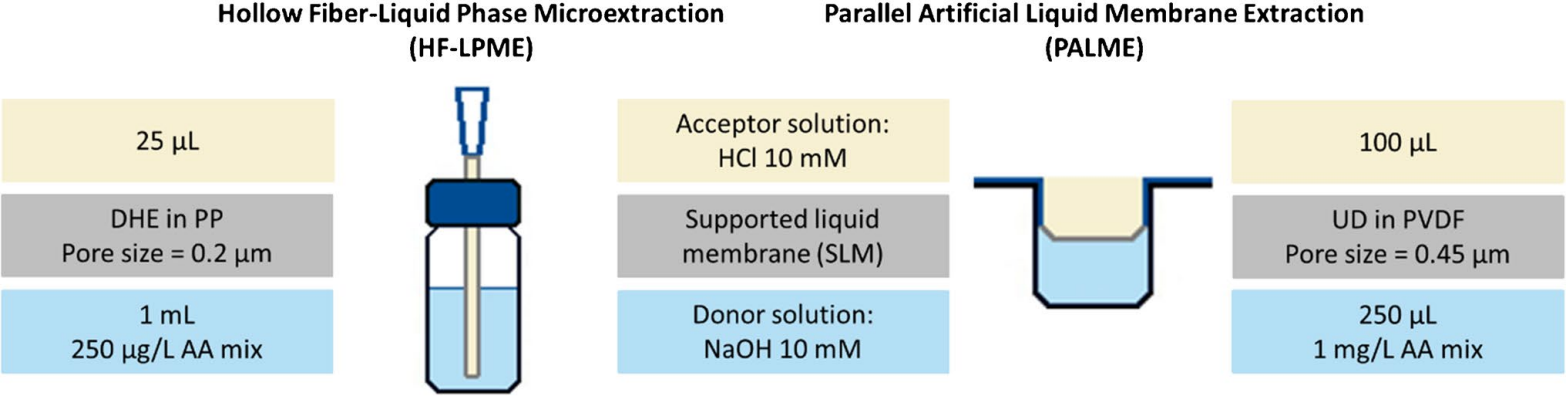
Schematic representation of the HF-LPME and PALME (one well) setups, including conditions used in this paper. Abbreviations: AA, aromatic amines; DHE, dihexylether; HCl, hydrochloric acid; NaOH, sodium hydroxide; PP, polypropylene; PVDF, polyvinylidene fluoride; UD, undecane

The setups used and described in the following sections can be seen in Fig. 1.

##### HF-LPME setup and procedure

The fiber used was a Q3/2 polypropylene membrane from Membrana (Wuppertal, Germany), with a pore size of 0.2 µm, an internal diameter of 1200 µm, and a wall thickness of 200 µm. The HF-LPME setup was prepared following Gjelstad et al. [15]. The fiber was cut into 2-cm-long pieces, one end was sealed together with pliers, and the other one was fixed to an approximately 2-cm piece of a Finntip 200 Ext pipette tip (Sigma-Aldrich) using a soldering iron (Supplementary Information, SI, Fig. S 1). The HF was then placed, through the lid’s septa (Fig. S 2), into a 2-mL vial containing the organic solvent for 3–5 s, in order to condition the fiber walls. Afterwards, 25 µL of the acceptor solution was added with a microsyringe (Hamilton Robotics, Bonaduz, Switzerland) into the lumen of the HF, using the pipette tip in the HF as a needle guide. Finally, the HF was placed into a vial containing 1.0 mL of the donor solution.

The vial with the extraction setup was shaken using a KS 260 control shaker (IKA, Staufen, Germany). After a set extraction time, the hollow fiber was directly removed from the donor solution. The acceptor solution was then carefully transferred into a 10-mL amber glass vial with a microsyringe. When multiple samples were extracted, first, all the fibers were taken out of the donor solution and placed into empty vials, and then, the acceptor solutions were collected.

##### PALME setup and procedure

Ninety-six-well plates with 0.5 mL or 1.25 mL wells from Agilent (CA, USA) were used as the donor plate and 96-well multiscreen-IP filter plates with polyvinylidene fluoride (PVDF) membranes, a pore size of 0.45 µm, and thickness of 100 µm from Merck (Darmstadt, Germany) were used as the acceptor plate.

The membrane was conditioned with 5 µL of organic solvent using a pipette (Eppendorf, Wesseling, Germany, Fig. S 3). Then, 250 µL or 1 mL of the donor solution was added into the donor plate and 100 µL of the acceptor solution was pipetted in the well of the membrane plate and sealed with a multi-plate sealing film (HS-300, Axygen Scientific, USA). Both plates were then clamped together and closed with the lid from the acceptor plate. The system was shaken using a KS 260 control shaker for a defined time, after which the plates were separated and the acceptor solution was collected with a microsyringe and transferred into a 10-mL amber glass vial.

##### Optimization experiments

A one factor at a time optimization approach was followed: after each parameter was optimized, the value that provided the best results was used for the subsequent optimizations. First, different organic solvents (DDA, 2O, UD, and DHE) were tested with both techniques. During those experiments, the rest of the parameters were kept constant: the samples (or donor solutions) were extracted for 45 min at 250 rpm with a 10 mM HCl (pH = 2) acceptor solution. Agitation speeds of 150, 250, and 350 rpm; extraction times of 15, 30, 45, 60, 75, min; and acceptor solutions with pH values of 1, 2, 3, and 4 were studied both with HF-LPME and PALME. A further optimization experiment using PALME was performed by testing 15, 20, 25, and 30 min, and 500 rpm in order to more precisely define the optimal extraction time and speed. HF-LPME was not studied further due to the outstanding capabilities of PALME in comparison. All measurements were done in triplicate.

#### Derivatization

Prior to derivatization, acceptor solution was added to the extracted sample until a final volume of 100 µL was reached to have constant starting volumes for derivatization and ensure comparability between samples.

The samples were derivatized following a procedure based on a diazotization and subsequent iodination reactions [16]. Into 100 μL of the extracted sample, 100 μL hydriodic acid (55%) and 200 μL sodium nitrite (50 g/L) were added and the samples were shaken for 20 min at 300 rpm, transforming the amine group of the aromatic amines into diazonium ions. To destroy the surplus of nitrite, 500 μL of sulfamic acid (50 g/L) was added, shaking subsequently for 45 min at 300 rpm. The samples were then heated in a water bath at 95 °C for 5 min to facilitate the substitution of the diazo group by iodine. To reduce the surplus of iodine, 250 µL of sodium sulfite (120 g/L) was added to the cooled down sample, which triggered an immediate discoloration of the initially brownish solution. Finally, 100 μL of alizarin red S (1% w/v) and 92 µL NaOH (10 M) were added to the samples to adjust the pH of the sample to 5.

The samples used for the optimization tests were derivatized automatically thanks to the PAL RTC from CTC Analytics AG (Zwingen, Switzerland). A few modifications were done to the procedure, such as vortexing the reagents before addition and the samples after reagent addition. For the method validation experiments, the derivatization was done manually due to the increased throughput needed, since with the PAL RTC only six samples could be derivatized at the same time, due to the six positions in the agitator.

### SPME

To enrich the iodinated derivatives before measuring, a DVB/PDMS SPME fiber with a thickness of 110 µm and a length of 10 mm from BGB Analytik Vertrieb GmbH (Rheinfelden, Germany) was used, in combination with an IP-deactivated SPME liner from Restek (Bad Homburg, DE).

The samples were pre-incubated at 60 °C for 10 min under agitation at 500 rpm, while the fiber was being conditioned in the SPME conditioning station for 8 min at 230 °C. The SPME fiber was then injected into the headspace of the vial (still at 60 °C and under agitation) for 25 min. Afterwards, the extracted analytes were desorbed into the GC-injection port for 5 min.

### Instrumentation

All analyses were performed by a Shimadzu GCMS system consisting of a GC-2010 Plus gas chromatograph coupled to a GCMS-QP2010 Ultra mass spectrometer from Shimadzu GmbH (Duisburg, Germany). Control of the GC–MS system was done with the GCMS Real Time Analysis software, Shimadzu GmbH. The system was connected to a PAL RTC autosampler, which was controlled with Chronos from Axel-Semrau (Sprockhövel, Germany). Separation of the analytes was achieved with a Rxi-5MS column (30 m, ID: 0.25 mm, film: 0.25 μm) from Restek. The septa used throughout all the experiments were AG3-Shimadzu septa from Macherey–Nagel (Düren, DE).

The temperature of the injection port was set to 230 °C. Helium (99.999% from Air Liquide, Krefeld, Germany) was used as the carrier gas with a constant column flow of 1 mL/min and a purge flow of 3 mL/min. The linear velocity was selected as flow control mode, and was set to 36.1 cm/s. The instrument was operated in splitless mode with a sampling time of 5 min, and using a split ratio of 10:1 afterwards. The initial oven temperature of 40 °C was held for 1 min, then increased to 230 °C with a 10 °C/min rate, and subsequently held for another minute, adding to 21 min total run time. At the starting temperature, and with the parameters aforementioned, the column head pressure was 49.7 kPa.

The MS interface and the ion source temperature were set to 230 °C. The solvent cut time was 5 min and the detector voltage was 1 kV. Full scan mode was used, and in order to achieve a better sensitivity, only the mass to charge ratios between m/z 74 and 470 were studied, using a scanning speed of 10,000 amu/s. The data were processed with the GCMS Post Run Analysis software from Shimadzu GmbH and evaluated using Excel (Microsoft).

### Data evaluation

The m/z ratios used as reference ions and those used for quantitation can be seen in the SI (Table S 1). The peaks were automatically integrated with the GCMS Post Run Analysis software (Table S 2). All peaks were visually checked for correctness and adjusted if necessary. Outliers were detected using the Dixon test [17]. To check for statistical differences between sample sets, Welch’s two-sided *t*-test or the two-variable *t*-test was used, depending on whether the variances of the two data sets were significantly different or not, which in turn was determined with Fisher’s *F*-test [17]. Recoveries for HF-LPME and PALME were based on the weekly control samples (2.5 mg/L aromatic amine mix) and calculated according to Gjelstad et al. [18]. More information, including the equations used, can be found in the SI.

The calibration curve and the limits of detection (LODs) and of quantification (LOQs) were calculated according to the DIN 32645 [19]. The repeatability (or intra-day precision) was calculated based on triplicates of the lower calibration point (100 ng/L) and based on the Eurachem Guide [20].

## Results and discussion

Thirteen aromatic amines were selected as model analytes due to their diverse chemical and physical properties, such as hydrophobicity (Log *P*) and pKa values. Furthermore, most of them have either been found in urine samples [3] and/or were successfully extracted by LPME from aqueous samples, such as industrial, environmental, and surface/tap water [21–25].

### Organic solvent optimization

#### HF-LPME

Four organic solvents (DDA, DHE, 2O, and UD) were selected based on literature [15, 21, 25], in order to study the influence of the organic solvent forming the SLM in the extraction process.

**Fig. 2:**
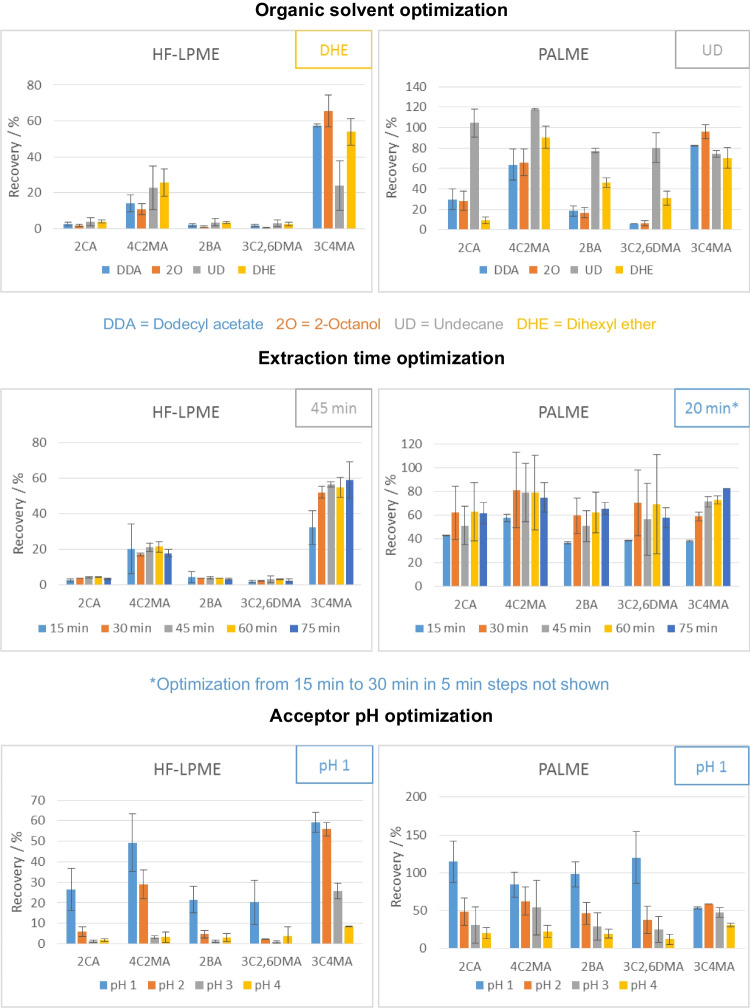
Optimization results of HF-LPME and PALME for a subset of the aromatic amines studied. Optimal values are shown at the top right of the corresponding graph. A one parameter at a time approach was used for optimization, starting with 45-min extraction at 250 rpm, and a pH = 2 acceptor solution

There was no solvent that consistently outperformed or underperformed in terms of recovery across the different analytes studied (Fig. 2, Fig. S 4). For example, 2O showed the best and worst recoveries for five and six of the analytes studied, respectively. DHE showed the best extraction efficiencies for seven of the studied analytes and showed good extraction efficiencies for the rest, which translated into a significantly higher geometric mean of the recoveries among the organic solvents tested. Therefore, it was the solvent used for further experiments.

There are a few studies in which aromatic amines were analyzed with different setups of HF-LPME (Table 2), and different solvents. In agreement with the results shown here, DHE was chosen as optimal solvent in the two papers in which it was tested [21, 25].

**Table 2:** Summary of parameters optimized for the analysis of aromatic amines with HF-LPME from literature and from this study

General information			Organic solvent		Stirring speed		Donor pH		Aceptor pH		Extraction time		Ref.
Type	Mode	Analytes	Tested	Optimal	Range tested (rpm)	Optimal (rpm)	[NaOH] tested (M)	Optimal [NaOH] (M)	[HCl] tested (M)	Optimal [HCl] (M)	Range tested (min)	Optimal (min)	
Static	HF^2^-LPME & HF^3^-LPME	A, 4NA, 2,4DNA, 2,6DC4NA	DHE, UD	DHE	–	200**	0.01–0.3	0.1	n.a	8	10–120	80	[21]
Static	HF^2^-LPME	4MA, 3NA, 2,3DMA, 4CA, 3,4DCA, 4ABP	4:6 to 8:2 O:MeOH	6:4 O:MeOH	800–1000	800	–	0.1	–	–	5–50	30 (20, 50)	[22]
Dynamic	HF^3^-LPME	3NA, 4BA, 4CA, 3,4DCA	1O, T	1O, T	–	1000	0.01–1	0.1	0.05–0.5	0.5	10–40	20	[23]
Static	HF^3^-LPME	3NA, 4BA, 4CA, 3,4DCA	1O, DHE, DAE	DHE	−, 200, 1000	1000	0.001–1	0.1	0.001–0.5	0.5	5–60	30 (50)	[25]
Static	HF^3^-LPME*	3CA, 3NA, 4BA	1O, T, B, X, EB, H	T	360–960	800	0.001–1	1	0.001–0.5	0.5	15–90 s/n.a	75 s/10	[26]
Static	HF^3^-LPME*	See Table 1	DDA, 2O DHE, UD	DHE	150–350	250	–	0.01	0.0001–0.1	0.1	15–75	45	This study
Static	PALME			UD	150–500	500	0.01–0.3	0.3		0.1		20	

When several values gave optimal results for different amines, the values not used in following experiments are presented in parentheses ()

*Extraction and back extraction were not performed simultaneously, but in two consecutive steps. **Agitation speed instead of stirring speed

Abbreviations: *1O*, 1-octanol; *2,3DMA*, 2,3-dimethylaniline; *2,4DNA*, 2,4-dinitroaniline; *2,6DC4NA*, 2,6-dichloro-4-nitroaniline; *3CA*, 3-chloroaniline; *3NA*, 3-nitroaniline; *3,4DCA*, 3,4-dichloroaniline; *4ABP*, 4-aminobiphenyl; *4BA*, 4-bromoaniline; *4CA*, 4-chloroaniline; *4MA*, 4-methylaniline; *4NA*, 4-nitroaniline; *A*, aniline; *B*, benzene; *DAE*, diamylether; *DHE*, di-n-hexyl ether; *EB*, ethylbenzene; *H*, n-heptane; *HCl*, hydrochloric acid; *HF*^*n*^*-LPME*, n-phase hollow fiber–liquid-phase microextraction; *MeOH*, methanol; *n.a.*, not available; *NaOH*, sodium hydroxide; *O*, octane; *PALME*, parallel artificial membrane extraction; *T*, toluene; *UD*, undecane; *X*, o-xylene

#### PALME

The same solvents were tested with PALME, and UD was either significantly better or similar to the other solvents for all the analytes (Fig. 2, Fig. S 5). The different optimal solvents found in comparison to HF-LPME could be due to the different thicknesses of the fibers/membranes (100 µm PALME and 200 µm HF-LPME) or the different materials of which they are made of (PVDE PALME and PP HF-LPME).

### Extraction time optimization

#### HF-LPME

Five points were tested at 15-min intervals, from 15 to 75 min. After 60- and 75-min extraction, an intensity loss was observed for most analytes (Fig. 2, Fig. S 6). A smaller amount of acceptor solution could be recovered from the lumen of the fibers at these extraction times, and it is therefore believed to have leaked through the pores of the HF, as previously reported by Gjelstad et al. [15]. This could explain the recovery decrease observed after 45 min, instead of the expected plateau. Therefore, 45 min was used as extraction time for HF-LPME.

The extraction times used in the literature studied (Table 2) ranged from 10 to 80 min, and both in Lin et al. [22] and in Zhao et al. [25], 30 min was used as a compromise between extraction speed and efficiency. The optimal time found in this research, 45 min, would be expected considering that lower agitation speeds would lead to higher extraction times needed (see the “Agitation speed optimization” section).

#### PALME

Because for most analytes already after 30-min extraction time a plateau was reached, a second experiment with shorter times was performed (Fig. 2, Fig. S 7). For the majority of the analytes, the maximum recovery was reached after 20 min, and therefore, that time was chosen for the following experiments.

A shorter extraction time was needed in comparison to HF-LPME, most likely due to the different geometry of the setup, e.g., the thinner membrane.

### Agitation speed optimization

#### HF-LPME

Three agitation speeds, namely 150, 250, and 350 rpm, were tested, and for most analytes, no significant differences could be observed (Fig. S 8). Due to the apparent instability of the setup at higher speeds, and to avoid bubble formation as reported by [23], 250 rpm was used for future experiments.

In most of the literature (Table 2), the donor solution was stirred with a magnetic stirrer. In this study, smaller sample volumes were used and placed in 1-mL vials, where standard stirrers would not fit, and therefore, the whole setup was agitated instead. Because of that, smaller agitation speeds were used, comparable to those used in Tao et al. [21], where the complete setup was also shaken.

#### PALME

With PALME, the maximum speed of the shaker (500 rpm) was studied in addition to the speeds discussed above. The results with 500 rpm showed an improvement of the extraction efficiencies and were therefore used for the remaining experiments.

### Acceptor pH optimization

#### HF-LPME

Gjelstad et al. recommend to use a pH 1 to 3 units below the pKa value of the analytes for the acceptor solution [36]. Because the analytes studied had pKa values between 0.7 and 5.0, the influence of the acceptor solution pH was tested from pH 1 to pH 4.

As expected, and in agreement with literature (Table 2), the lower pH showed the best recoveries, with pH 1 and 2 showing significantly better results than pH 3 and 4 for most of the analytes (Fig. 2, Fig. S 10). Furthermore, pH 1 showed significantly better results than pH 2 for 2CA, and 2BA. This can be explained by the fact that these compounds have the lowest pKa values—after 2,6DCA—among the analytes studied (see Table 1), and therefore, a lower pH is needed to successfully trap the analytes in the acceptor solution. For 2,6DCA, no significant difference could be observed, probably due to the extremely low pKa of this analyte (0.7), which would indicate even lower pH values are needed.

#### PALME

A similar trend of increased recoveries with lower acceptor pHs was also observed with PALME, although not as extreme as with HF-LPME (Fig. 2, Fig. S 11). This could be explained by the higher recoveries already observed with higher pH values. Nonetheless, pH 1 showed the best results and was chosen for the following experiments.

### Optimized extraction techniques comparison

HF-LPME presents two major disadvantages. On the one hand, it is a much more labor-intensive setup. As it is not commercially available, it needs to be assembled manually, which not only takes time, but also can introduce small variations in the fibers that could have an influence on the recoveries observed. Furthermore, it is more mistake prone, as steps like insertion or removal of the acceptor solution into/from the lumen of the hollow fiber are much more sensitive: the fiber can easily break, or different size droplets can be left behind in the lumen. Moreover, the recoveries observed were much smaller compared to PALME. The smaller recoveries observed also contribute to small variations having a bigger impact, and could explain the fact that the RSDs observed with HF-LPME are generally higher than with PALME.

HF-LPME could be further optimized, for example, by trying different types of carriers and concentrations. However, the results obtained with PALME not only offer the advantage of significantly better recoveries, but also a much less labor-intensive and less error-prone design. Therefore, PALME is recommended over HF-LPME and was used for the validation experiments.

Before the PALME validation, a few further optimization experiments were done (not shown), where a higher donor pH (12 vs 13.5), more donor volume (0.25 vs 1 mL), the addition of an organic modifier (0 vs 25% methanol) in the acceptor solution, and an increased temperature (40 °C vs room temperature) were studied. Positive effects were observed for a higher donor pH (13.5) and more donor volume (1 mL), so these conditions were used for the validation experiments.

### Method validation

The results obtained in this study are generally comparable with literature (Tables 3 and 4). The selection of the linear range, 100–1200 ng/L, was based on the expected concentrations of AA in urine samples [27–32] and preliminary studies with this setup, and it includes the lowest calibration point reported for LPME measurements, namely 500 ng/L (Table 4). Furthermore, the donor volumes were set to ≤ 1 mL, which is relatively low in comparison to the literature found where LPME is used for the analysis of aromatic amines [21–25]. This value was chosen in order to study if LPME can be suitable for the analysis of valuable archived samples and it is between 4 and 100 times smaller than the donor volumes studied in literature. Despite that, the results obtained were satisfactory, with correlation coefficients of 0.991–0.999 for nine AA. Furthermore, the RSD based on peak areas and the repeatability (or intra-day precision) of the lowest calibration point (100 ng/L) were below 20%. The LODs were calculated based on *S*/*N* = 3 for an easier comparison with literature, and based on the calibration curve for a more accurate approach. The LODs obtained in this study based on *S*/*N* (100 ng/L, *n* = 3, root mean square calculation method, standard smoothing: 1 time, 1 s width) are 5 to 6500 times smaller than those reported in literature. The *S*/*N* approach offers a significant disadvantage, which is that the results can vary significantly based on the concentration point and method used for its calculation, and to which extent peak smoothing was applied, and unfortunately, these parameters are usually not reported. Therefore, the LODs based on the calibration curve as described by [24] are also presented. Even when using the more conservative approach based on the calibration curve, the LODs obtained in this study are some of the lowest reported so far for AA with LPME techniques. According to AA concentrations found in literature [27–32], these LODs should generally be sufficient to successfully analyze real samples. Such small LODs and the use of small donor volumes are especially critical when analyzing samples with limited availability where miniaturization is needed, like archived samples. Finally, two real samples were measured: 26DMA could be detected in both samples and 2MA could be quantified in one sample with 243 ng/L and detected in the other. The reason for most target analytes not being detected in these samples might be due to the smoking topography of the donors.

**Table 3:** Figures of merit of the aromatic amines (AA) studied where the regression coefficient (*R*^2^) was > 0.99, and the concentrations observed in two real samples of smokers

AA	*R*^2^	LOD (ng/L)	LOQ (ng/L)	RSD (%)	Sample	
		*S*/*N*/C.C.	*S*/*N*/C.C.		OS1 (ng/L)	OS2 (ng/L)
2MA	0.996	3/45	10/155	3	< LOQ (C.C.)	243
3C4FA	0.993	7/62	24/208	7	n.d	n.d
2CA	0.994	3/55	11/186	2	n.d	n.d
4EA	0.992	4/71	15/241	4	n.d	n.d
26DMA	0.998	6/35	19/122	5	< LOQ (C.C.)	< LOQ (C.C.)
2BA	0.993	12/60	39/203	12	n.d	n.d
4C2MA	0.994	3/57	9/193	2	n.d	n.d
246TMA	0.991	3/75	11/254	11	n.d	n.d
3C26DMA	0.996	12/45	41/156	7	n.d	n.d

Limits of detection and quantification (LOD and LOQ) were calculated based on signal to noise ratios of the lowest calibration point (100 ng/L, *n* = 3, root mean square calculation method, standard smoothing: 1 time, 1 s width), *S*/*N* = 3 and 10, respectively (left value), and based on the calibration curves obtained as described by [17] (C.C., right value). The relative standard deviation (RSD) was calculated based on the peak areas observed at the lowest calibration point (100 ng/L, *n* = 3)

**Table 4:** Figures of merit of most recent literature regarding the analysis of aromatic amines with LPME

Linear range (µg/L)	Regression coefficient (*R*^2^)	RSD (%)	LODs (µg/L)	Reference
5–200	0.995–0.999	n.a	0.5–1.5	[21]
5–240	0.992–0.997	4–7	2.1–4.8	[22]
100–10,000	0.997–0.999	7–14	8–20	[23]
0.5–500	0.992–0.999	5–7	0.05–0.10	[25]
0.5–1000	0.998–0.999	4–4	0.05–0.1	[26]
0.1–1.2	0.991–0.999	2–12	0.003–0.01 C.C.: 0.03–0.7	This study

Ranges reported correspond to the minimum and maximum values from different analytes. The relative standard deviation (RSD) was calculated based on the peak areas observed. The limits of detection (LODs) were calculated based on *S*/*N* = 3. In this study, the LODs were also calculated based on the calibration curves obtained (C.C.) according to [19]

A few AA could not be successfully analyzed. It is believed that 2,6DCA was mostly trapped in the donor solution and was not successfully extracted into the acceptor solution due to its extremely low pKa value (0.71 [13]). A, AAP, and 2,4DMA most likely had too low of a log *P* value (0.9, 1.6, and 1.7 [14]) and were discriminated by the SLM, as previously reported by [33]. An acceptor solution with a lower pH value could improve the extraction of analytes with low pKa values, and the use of ion-pair reagents could help with polar substances as described by Gjelstad [9].

## Conclusion

The optimized HF-LPME and PALME were compared, and PALME showed significant advantages, not only due to its simpler and less error-prone setup, but also due to the significantly higher recoveries observed. PALME was proven a very successful extraction technique, providing high enrichment of the AA and LODs in the nanograms per liter range, comparable or lower than those found in literature. Furthermore, compared to LLE, it has the extra benefit of being a greener technique, thanks to the significantly lower volumes of organic solvents needed. Moreover, the PALME setup is disposable, minimizing carry-over and the need for cleaning/regeneration, and thanks to the extra physical barrier, they typically extract less matrix interferences, making it an ideal technique for complex biological matrixes such as urine. Because of the low urine volume needed, this technique would also be suitable for the analysis of archived samples, such as those of completed medical studies, where the available sample volume is limited. And because of the high throughput possible, the method described here could be used in the future for a comprehensive study with different types of donors.

In this study, the PAL RTC was used for derivatization and SPME extraction. Because of the complexity of the automated system, including the RTC Pal and SPME, a similar setup may not be available in all routine laboratories, limiting its applicability. However, automation is considered a key step towards green chemistry [34], and offers multiple benefits, including minimized human intervention and errors and increased reproducibility, which in many cases will outweigh the costs. Further automation would be possible thanks to multichannel pipettes or pipetting robots [35]. Although there is no commercially available PAL RTC module for PALME, this autosampler could be used for a more automatic PALME by setting up an external shaker as a new module. The donor plate could be covered with sealing foil instead of the plastic cover so that it could be easily pierced by the autosampler. The donor, organic solvent, and acceptor addition, and the clamping of the plates should still be done manually, since there is no tool available that could perform that task. A main drawback of this semi-automatic approach is that the extraction could not be stopped by separating the plates. With 10 s per sample, it would already take over 15 min to place all 96 acceptor solutions into new vials. Depending on the analytical requirements, this could be accounted for by the use of internal standards, but further research would be needed. An alternative would be to do the extraction separately, and put only the acceptor plate into the autosampler. That way the separation is halted for all samples simultaneously. Because of the availability of a pipette tool for the PAL RTC, the risk of contamination when transferring the acceptor solution into vials can still be kept low. If automatic derivatization is needed, an agitator with more positions would be beneficial for higher throughputs.

## Supplementary Information

Below is the link to the electronic supplementary material.

Supplementary file1 (DOCX 1.91 MB)
