# Supplementary material for: Liquid-phase microextraction of aromatic amines: hollow fiber–liquid-phase microextraction and parallel artificial liquid membrane extraction comparison
Source: Anal Bioanal Chem. 2023 Feb 23;415(9):1765–76. doi: 10.1007/s00216-023-04579-w (PMC9992073; doi:10.1007/s00216-023-04579-w)
Supplement: Supplementary file 1 — Supplementary file1 (DOCX 1.91 MB) [file 216_2023_4579_MOESM1_ESM.docx]

**Supplementary Information**

For the paper **Liquid-phase microextraction of aromatic amines: hollow fiber–liquid- phase microextraction and parallel artificial liquid membrane extraction comparison,** by Nerea Lorenzo-Parodi, Wiebke Kaziur-Cegla, Astrid Gjelstad, Torsten C. Schmidt.

**Hollow fiber assembly**

The fiber was cut into approximately 2 cm long pieces (Fig. S 1, A and B), from which one end was clamped together (Fig. S 1, C and D) before being glued to a piece of a pipette tip using a soldering iron (Fig. S 1, E, F and G) to get the final Hollow fiber (Fig. S 1, H).


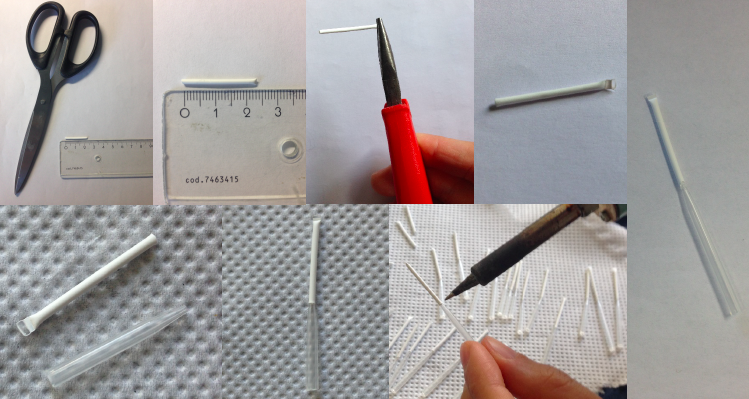


**A B C D H**

**E F G**

Fig. S 1. Pictures taken during the preparation process of a hollow fiber. A: Scissors, ruler and approximately 2 cm long fiber. B: Cut fiber. C: Sealing fiber with pressure at one end using clamps. D: Fiber with one closed end. E: Clamped fiber and pipette tip piece. F: Pipette tip inserted in fiber. G: Soldering the fiber to the pipette tip. H: Final hollow fiber assembly with pipette tip as a needle guide.

The hollow fibers were fixed in the vial caps to avoid risk of losing the acceptor solution. First, the septum was perforated with a needle (Fig. S 2, B), and the hole was widened with a piece of a pipette tip, called “guiding tip” hereon (Fig. S 2, C). The pipette tip piece of the hollow fiber was then connected to the guiding tip (Fig. S 2, D), so that the hollow fiber could be pulled through the septum (Fig. S 2, E). Then the guiding tip was separated from the hollow fiber (F). The lid was finally screwed on top of a 2 mL amber glass vial (Fig. S 2, G).


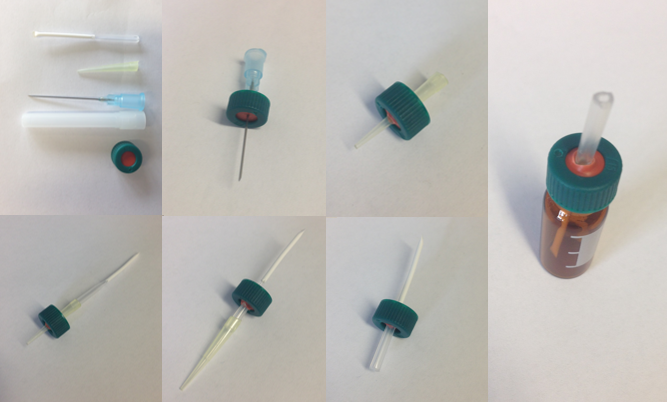


A B C

D E F G

Fig. S 2. Fiber assembly into a vial. A: Tools required for the process - from top to bottom: hollow fiber, pipette tip, needle, housing of needle, vial screw cap. B: Septa pierced with needle. C: pipette tip penetrated through the hole in the septa. D: Hollow fiber attached to the pipette tip. E: Hollow fiber pulled through the septa. F: Hollow fiber in its final position in the septa. G: Hollow fiber assembled into a 2 mL vial.

**PALME set-up and procedure**

The PALME workflow followed was adapted from [1] (Fig. S 3). The 96-well plates used as the donor plate can be seen in Fig. S 3 - 1 and 2, and the 96‑well filter plates used as the acceptor plate in Fig. S 3 - 3, 4 and 6.


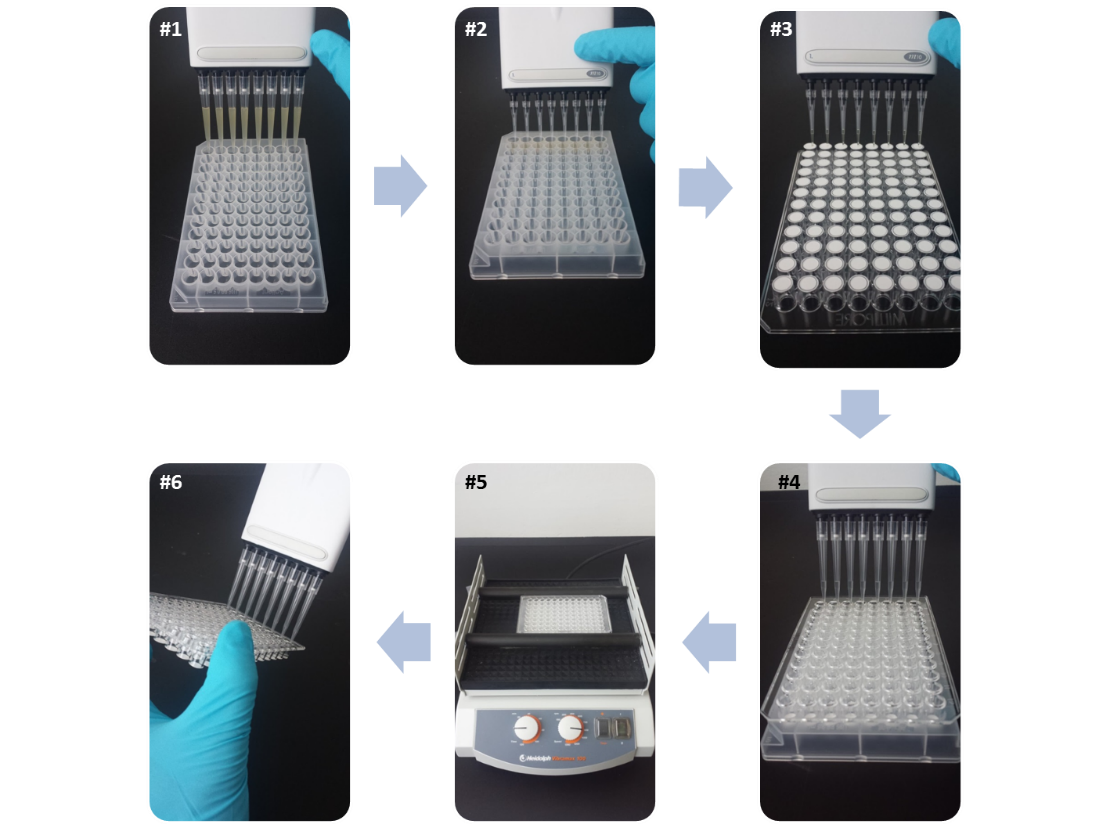


Fig. S 3. Typical PALME workflow. Step #1: pipetting samples, Step #2: pipetting internal standards and buffer, Step #3: pipetting SLMs, Step #4: pipetting acceptor solutions, Step #5: clamping the plates together and shaking of the set-up, Step #6: transfer of acceptor solutions. Reproduced from [1] with permission.

**Data evaluation – Peak integration parameters**

During the sample preparation, all aromatic amines were derivatized into their corresponding iodinated versions. The iodinated compounds were then separated and detected via GC-MS. The quantifier and qualifier ions used for their detection, generally corresponding to the molecular ion and the loss of iodine, can be seen in Table S 1.

Table S 1. Mass spectrometric parameters used for the detection of the derivatized aromatic amines, including the quantifier and the qualifier ion of each analyte studied.

| Analyte | Iodinated aromatic compound | Quantifier ion  (m/z) | Qualifier ion  (m/z) |
| --- | --- | --- | --- |
| A | Iodobenzene | 204 | 77 |
| 2MA | 1-Iodo-2-methylbenzene | 218 | 91 |
| 3C4FA | 3-Chloro-4-fluoro-1-iodobenzene | 256 | 129 |
| 2CA | 2-Chloro-1-iodobenzene | 238 | 111 |
| 4EA | 4-Ethyl-1-iodobenzene | 232 | 217 |
| 2,6DMA | 2,6-Dimethyl-1-iodobenzene | 232 | 105 |
| 2,4DMA | 2,4-Dimethyl-1-iodobenzene | 232 | 105 |
| 4C2MA | 4-Chloro-1-iodo-2-methylbenzene | 252 | 125 |
| 2BA | 2-Bromo-1-iodobenzene | 282 | 155 |
| 2,4,6TMA | 1-Iodo-2,4,6-trimethylbenzene | 246 | 119 |
| 2,6DCA | 2,6-Dichloro-1-iodobenzene | 272 | 145 |
| 3C2,6DMA | 3-Chloro-2,6-dimethyl-1-iodobenzene | 266 | 139 |
| 2NA | 2-Iodonaphthalene | 254 | 127 |

The chromatograms where automatically integrated using the GCMS Post Run analysis software and the parameters described in Table S 2.

Table S 2. Settings selected for the automatic peak integration with the GCMS Post run Analysis software.

| Peak integration | | Peak Identification | |
| --- | --- | --- | --- |
| Slope | 100 /min | Window for target peak | 5 % |
| Width | 3 s | Window for reference peak | 5 % |
| Min. Area | 4000 | Default Band time | 0.8 min |
| Base | Area | Reference Ion Mode | Relative |
| Smoothing | None | Ref. Ions based on | Spectrum |
| Processing time | 0.8 min | Correction of Ref. Ions ratio | No Change |

**Data evaluation – Dixon Q test**

Dixon Q test was use to find and exclude outliers from further calculations. Q_crit_ was taken from [2], as shown in Table S 3, and Q_calc_ was calculated with Equation 1. If Q_calc_>Q_crit_, the tested value was an outlier and was neglected during data evaluation. This test was applied to all measured samples.

| $Q_{calc}=\frac{\left\vert x_{2}-x_{1} \right\vert}{\left\vert x_{n}-x_{1} \right\vert}$ | Equation 1 [3] |
| --- | --- |

Table S 3. Critical values of Dixon’s test (Q) for a confidence interval of 90 % and sample sizes from three to six [2].

| Sample size N | Q_crit_ 90 %, α=0.1 |
| --- | --- |
| 3 | 0.941 |
| 4 | 0.765 |
| 5 | 0.642 |
| 6 | 0.560 |

**Data evaluation – Fisher’s F-Test, two-variable t-test and Welch’s two sided t-test**

The Fisher’s F-Test (Equation 2) was used to check if the variances of both data groups (s_1_^2^, s_2_^2^) were significantly different.

| $F=\frac{s_{1}^{2}}{s_{2}^{2}}$ | Equation 2 [3] |
| --- | --- |

If the calculated F-value was smaller than the tabulated one (Table S 4), the sample variances were not significantly different, and the two-variable t-test could be used.

Table S 4. F-quantiles for 95 % confidence interval for the different degrees of freedom f_1_ and f_2_ [3].

| f_2_ | f_1_=1 | 2 | 3 | 4 | 5 |
| --- | --- | --- | --- | --- | --- |
| 1 | 161 | 200 | 216 | 225 | 230 |
| 2 | 18.51 | 19.00 | 19.16 | 19.25 | 19.30 |
| 3 | 10.13 | 9.55 | 9.28 | 9.12 | 9.01 |
| 4 | 7.71 | 6.94 | 6.59 | 6.39 | 6.26 |
| 5 | 6.61 | 5.79 | 5.41 | 5.19 | 5.05 |

For the two-variable t-test, the means of each data set were compared using *Equation 3*, where n_1_ and n_2_ are the numbers of parallel determinations for the two sets of samples and s_d_ is the weighted averaged standard deviation, which was calculated using *Equation 4*.

| $t=\frac{\left\vert\bar{x}_{1}-\bar{x}_{2} \right\vert}{s_{d}}\sqrt{\frac{n_{1}n_{2}}{n_{1}+n_{2}}}$ | *Equation 3 [3]* |
| --- | --- |
| $s_{d}= \sqrt{\frac{\left( n_{1}-1 \right)s_{1}^{2}+\left( n_{2}-1 \right)s_{2}^{2}}{n_{1}+n_{2}-2}}$ | *Equation 4 [3]* |

If the calculated F-value was bigger than the tabulated one, the general t-test, after Welch, was applied (Equation 5) [3].

| $t=\frac{\left\vert\bar{x}_{1}-\bar{x}_{2} \right\vert}{\sqrt{\frac{s_{1}^{2}}{n_{1}}+\frac{s_{2}^{2}}{n_{2}}}}$ | Equation 5 [3] |
| --- | --- |

The calculated t-value (with either of the methods) was then compared to the tabulated t-value for 95 % significance. If the calculated t-value was smaller than the tabulated one (Table S 5), the sample sets were not significantly different [3].

Table S 5. Quantile of the t-distribution for the significance level of 95 % and the different degrees of freedom, f [19].

| f | t 95 % |
| --- | --- |
| 2 | 2.920 |
| 3 | 2.353 |
| 4 | 2.132 |
| 5 | 2.015 |

**Data evaluation – Recovery**

The recovery calculations were based on the peak intensities as described in *Equation 6*.

| $R= \frac{{Intensity}_{LPME sample}}{{Intensity}_{Control sample}} \times100 \%$ | *Equation 6* |
| --- | --- |

**Organic solvent optimization**


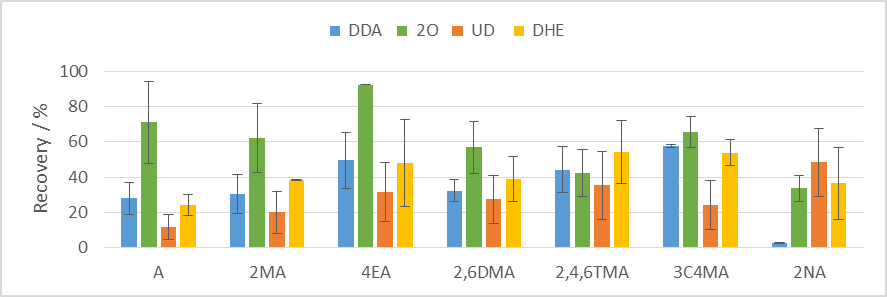


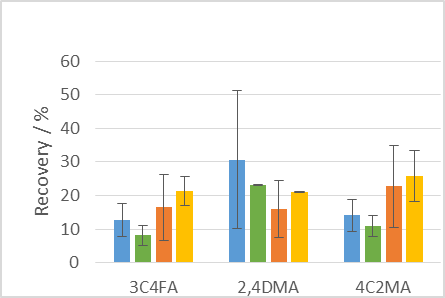

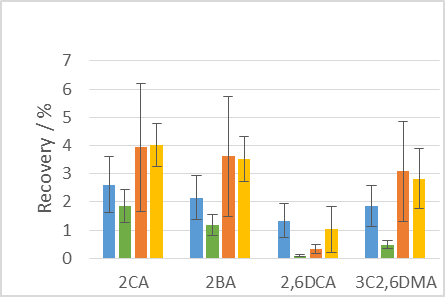


Fig. S 4. Influence of organic solvents during HF-LPME on the recovery of the aromatic amines studied. With DDA = dodecyl acetate, UD = undecane, 2O = 2-octanone, and DHE = dihexylether. The experiments were done in triplicate and outliers determined by the Dean Dixon outlier test were not considered.


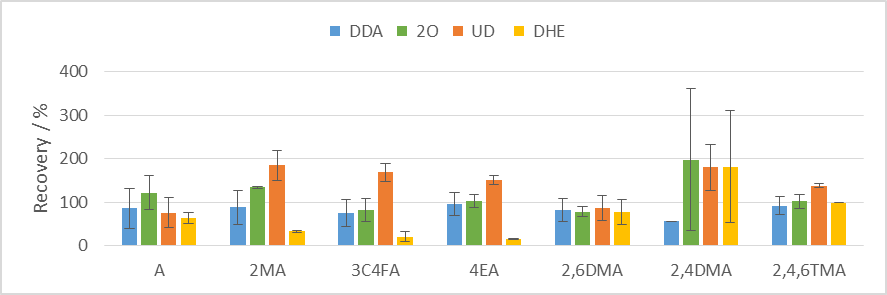


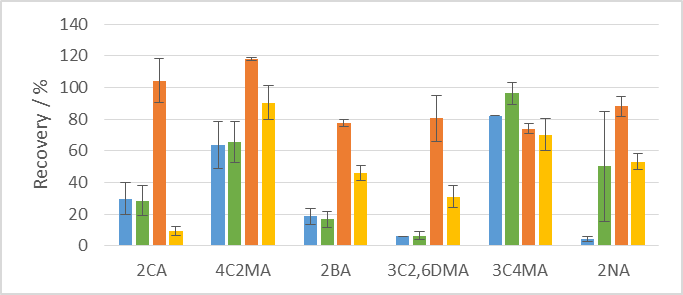

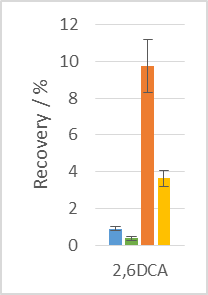


Fig. S 5. Influence of organic solvents during PALME on the recovery of the aromatic amines studied. With DDA = dodecyl acetate, UD = undecane, 2O = 2-octanone, and DHE = dihexylether. The experiments were done in triplicate and outliers determined by the Dean Dixon outlier test were not considered.

**Extraction time optimization**

Fig. S 6. Influence of extraction times during HF-LPME on the recovery of the aromatic amines studied. The experiments were done in triplicate and outliers determined by the Dean Dixon outlier test were not considered. Duplicates are presented for 45 min, as the hollow fiber detached from the needle guide, and no acceptor solution could be collected.


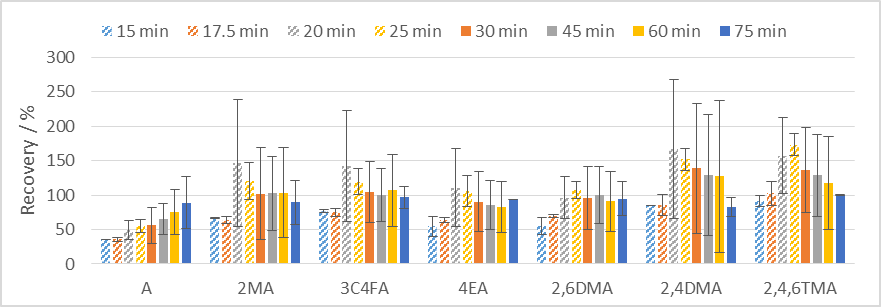


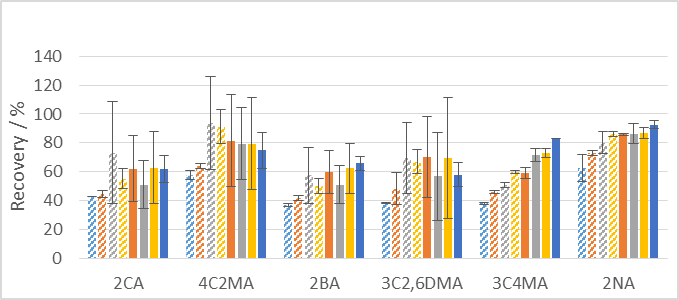


Fig. S 7. Influence of extraction times during PALME on the recovery of the aromatic amines studied. The experiments were done in triplicate and outliers determined by the Dean Dixon outlier test were not considered. The patterned columns were measured in a separate experiment.

**Agitation speed optimization**

Fig. S 8. Influence of agitation speeds during HF-LPME on the recovery of the aromatic amines studied. The experiments were done in triplicate and outliers determined by the Dean Dixon outlier test were not considered.

Fig. S 9. Influence of agitation speeds during PALME on the recovery of the aromatic amines studied. The experiments were done in triplicate and outliers determined by the Dean Dixon outlier test were not considered. 500 rpm results are not shown as they were compared to 250 rpm in a different experiment.

**Acceptor pH optimization**


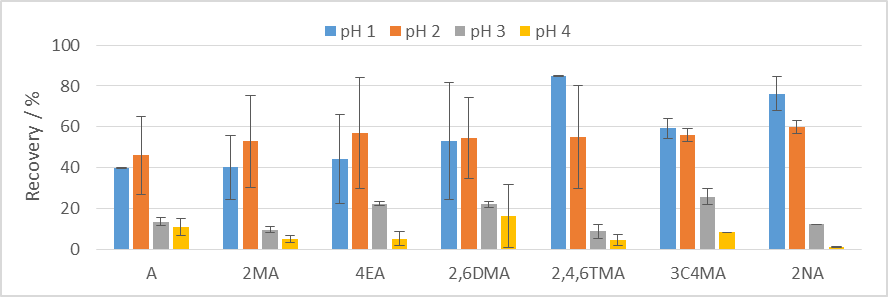


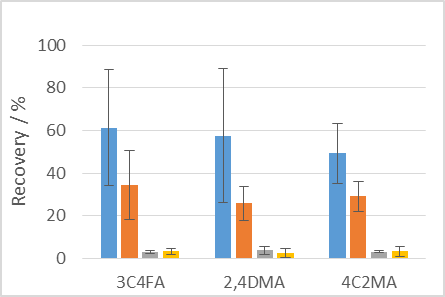

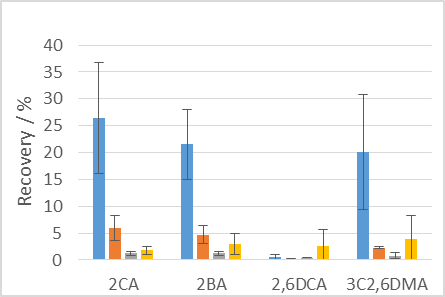


Fig. S 10. Influence of acceptor pH during HF-LPME on the recovery of the aromatic amines studied. The experiments were done in triplicate and outliers determined by the Dean Dixon outlier test were not considered.


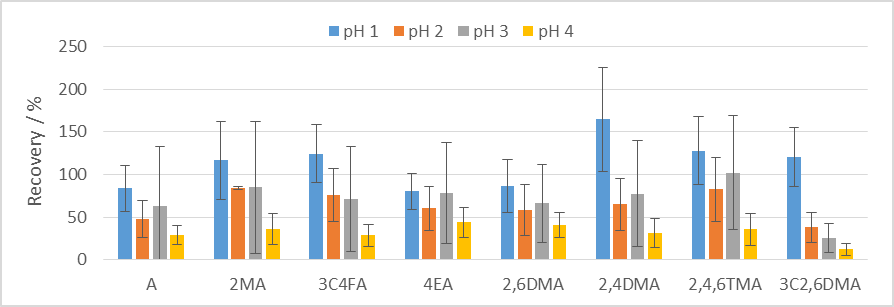

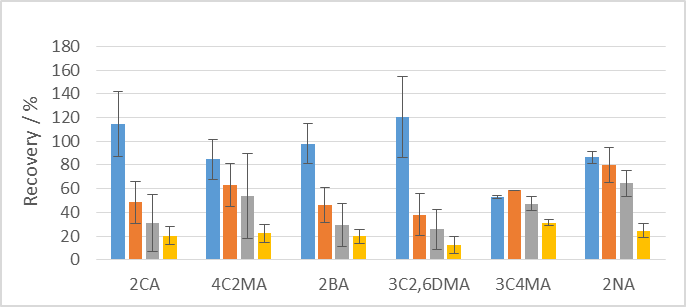

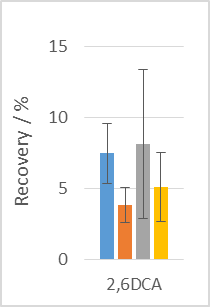


Fig. S 11. Influence of acceptor pH during PALME on the recovery of the aromatic amines studied. The experiments were done in triplicate and outliers determined by the Dean Dixon outlier test were not considered.

**References**

1. Gjelstad A, Andresen AT, Dahlgren A, Gundersen TE, Pedersen-Bjergaard S. High-throughput liquid-liquid extraction in 96-well format: Parallel artificial liquid membrane extraction. LC GC Europe. 2017;30(1):10-7.

2. Rorabacher DB. Statistical Treatment for Rejection of Deviant Values: Critical Values of Dixon’s “Q“ Parameter and Related Subrange Ratios at the 95% Confidence Level. Analytical Chemistry. 1991;63(2):139-46.

3. Otto M. Chemometrics Statistics and Computer Application in Analytical Chemistry. 3 ed. Weinheim, Germany: Wiley-VCH; 2016.
